# The Jaffray Suburban Hospital, Birmingham

**Published:** 1889-11-02

**Authors:** 


### The Hospital Workshop.

### No. XIII.—THE JAFFRAY SUBURBAN HOSPITAL, BIRMINGHAM.

(BY OUR SPECIAL COMMISSIONER.)

This branch institution was founded with the view of relieving the pressure on the General Hospital. Its story is briefly recorded on a marble tablet in the entrance hall :

"This hospital, standing in eight acres of freehold land, was built and equipped by John Jaffray, J.P., D.L., of Edgbaston, Birmingham, and was opened by H.R.H. the Prince of Wales, November 27, 1885. 'To be a help to the helpless.'"

Two hundred and eighteen patients were treated here in 1888. The report says: "There are no 'privileges' attached to admission—once in the General Hospital, and a fitting patient for transfer to Gravelly Hill, no ticket is necessary, and the stay there is not limited, excepting by a return to health or some other result of the case. One of the patients recently discharged occupied a bed for about two and a-half years—a period of rest and comfort unattainable in an ordinary hospital, and for which he was deeply grateful." That, however, is an exceptional case, for six months is the average limit of residence. The endowment fund from various sources amounts to £38,000. Last year's expenditure exceeded the income by £237 8s. 5d.

The "Jaffray" is situated at Gravelly Hill, a delightful suburb of Birmingham, and can be reached by train or by tram, either of which puts one down about a quarter of an hour's walk from the grounds. Passing the lodge gate, a neatly-kept winding drive leads to the hospital. On either side are borders of well-trimmed turf, brightened here and there by beds of flowers, and planted with firs and other ornamental trees.

The building is of red brick, and consists of two wings joined by a cross building in which are the living rooms of nurses and the resident staff. The entrance-lobby is square and of moderate size. It is floored with deep red tiles, an eight-day clock stands in the corner, and the visitor is at once impressed with a sense of the solidity and unpretentious comfort that pervades the whole place. There are several paintings on the walls ; a large canvas by Morley depicts a scene from "Romola"; a second is a copy from Turner, and among the others is a fine portrait of Mr. Jaffray, the founder, by the late Frank Holl. On either side are residential rooms, and the door in front leads into the main corridor, running along the whole length of the building. On the occasion of a late visit, Mr. J. D. Ballance, the resident medical officer, kindly afforded the information upon which this sketch has been chiefly founded.

The male surgical was the first ward entered. It contains twelve beds and a cot, and since all the wards are built and fitted out on the same plan, a detailed description of one will suffice for all. The floors are of polished oak, the walls blue, and the light from the ample windows being skilfully broken by ruby-tinted top panes. In the centre stands a good-sized table, the before-mentioned cot, and a large, comfortable couch for convalescents. Ventilation is well provided for, the inlets are the windows and valves, made of talc, let into the walls, and admitting air from outside. Outlets are provided by gratings over the door leading to an extraction shaft and by the open fireplaces, which, it may be noted, are of modern construction, and set with Minton tiles. Heating is effected chiefly by means of hot-water pipes. The bedsteads, curiously enough, are of the same old-fashioned four-poster type that is in use at the "General." They are inconvenient for nursing and for surgical purposes, and seem somewhat out of place in an advanced modern hospital. The counterpanes are of light blue with a figured pattern, and match well with the walls, while a "Nightingale," or red flannel wrap for the shoulders is folded neatly and placed across the foot of each bed. Each patient has a most ingenious locker, forming, when shut, a convenient seat. In the upper part are several shelves closed in by a door that can be let down over the seat to form a table. The lower part is a clothes-cupboard. Yonder is a boy of ten, who has suffered for several years from hip-joint disease. Let us see what he has on his shelves. Tea, butter, sugar, cocoa, and—a money-box. He says the latter contains one shilling and twopence, which he frankly owns he is going to spend in buying sweets. The boy occupies an endowed bed, of which there are seven in the hospital, amongst them one bequeathed by the late Miss Rylands. Coming back to our inspection, we notice that the straight lines of the ceiling are broken by cross-rafters, and this seems to be the only structural difference between the downstair and the upstair wards. On the mantelpiece are several brass vessels, which the patients take a pride in keeping polished to the highest pitch of brilliancy. Scattered around are the usual ornamental features of ward life—pictures, plants, flowers, and a bird or two. There is also a movable piano, which can be wheeled about as required when concerts are given by the residents or by the Kyrle Society. But the chief charm is the surrounding atmosphere of country life ; the quiet, broken only by such sounds as the crowing of a distant cock or the lowing of cattle ; the fresh green of grass and trees that can be seen from the windows. The contrast of all this freshness and quietude must be almost like the revelation of a new world to those who have been bred in the crowded alleys of smoky Birmingham. Yet it is a curious fact that patients, especially the younger ones, are always anxious to get back to their own homes. Did the writer of "Home, sweet home," ever study life in a hospital? At any rate, a better illustration of the strong magnetic force of the "never so humble" could hardly be found than the yearning of these little ones for their ancestral alleys. On one side of the ward entrance is a room containing a special bed. A glance inside revealed the hospital kite, tall as a grenadier, leaning stiffly against the wall. The ward-kitchen and other arrangements are convenient, and it may be specially remarked of the bath that it is sunk in the floor to within a foot of the rim, a capital plan when dealing with helpless patients. By means of a blanket a couple of attendants can lower or raise their charge with comparative ease.

Upstairs are some striking pictures, among which may be noticed a sea piece by F. W. Hayes, "Crossing the Brook," a picture full of skilfully handled contrasts by H. H. Emmerson ; and a powerful rendering of a "Scotch Mist," by S. J. Barnes. Let us now look at one or two cases.

Here is a lead-poisoning case. It is that of a lad of sixteen who worked for two years in a factory "mixing paint." He was taken into the "General" and under treatment had almost recovered, when, unfortunately, he was attacked with scarlet fever, which brought on the lead symptoms worse than ever. He is much paralysed ; he cannot walk, and has double "wrist-drop." His present treatment consists of a thorough course of massage and electricity, under which it is to be hoped he may again recover.

In the women's wards are a number of children. One of them, but five years of age, was sent back to the "General" to have her leg amputated just below the knee, and is now convalescing here. Poor girl! She will have an excellent stump for an artificial leg, a statement that may sound somewhat callous, but it means a good deal for the poor girl's future comfort in life. Her playmate in the next bed, a hip-joint case, has a large wax doll in a pink dress, and a hat of the same colour that partly covers a rich profusion of yellow hair. Dolly's cheeks of cherry red make a sad contrast with those of her little owner. Both these children want to go home.

Our visit was on a Friday, the day on which the ambulance comes over from the "General" with fresh cases. To-day there are two admissions, the one a man recovering from lead-poisoning, and the other a young woman who is suffering from diseased bone in the leg. Patients who get out into this pure air and healthy building, where they are provided with every care and comfort, are indeed fortunate.

